# The Emotions in Cultural-Historical Activity Theory: Personality, Emotion and Motivation in Social Relations and Activity

**DOI:** 10.1007/s12124-021-09615-x

**Published:** 2021-04-29

**Authors:** Ian Burkitt

**Affiliations:** School of Social Sciences, University of Bradford, Bradford, BD7 1DP UK

**Keywords:** Cultural-historical activity theory, Emotion, Feeling, Motivation, Personality

## Abstract

This article conceptualizes human emotion as formed within the cultural and historical development of each individual within their society, using theories of emotion created within cultural-historical activity theory (CHAT). It examines how the process of the cultural formation of emotion takes place as part of the social development of the personality, intimately tied in with motivation, need and desire. In doing this I review ideas about emotion in the work of the two founding thinkers of cultural-historical activity theory, L. S. Vygotsky and A. N. Leontyev; however, for different reasons their work has its limits in creating a contemporary understanding of emotion. Moving forward, I draw on more recent work on emotions in the field of CHAT, as well as on the work of M. M. Bakhtin, to create a more embodied understanding of the cultural-historical formation of the emotions as part of the dynamics of personality as a whole, including its conscious and unconscious elements.

In recent years the subject of emotions has become more prominent amongst those who work in the field of cultural historical psychology and activity theory. Most of this work in the tradition of cultural-historical activity theory (CHAT)^1^ takes as its starting point the thinking of the two seminal figures in this field, L. S. Vygotsky and A. N. Leontyev. Although both differed in the orientation and detail of their respective approaches they were agreed on one thing: that in basing their scientific studies in the historical materialism of Marx and Engels, humans were to be understood not solely as natural beings but primarily as social and historical beings. In the history of the social relations of production, humans have transformed the natural environment into a socially produced mode of life (Marx & Engels, 1970) and, in the process, have transformed their own nature to become individuals who are created through social and historical development rather than in biological maturation alone. For Vygotsky (1987a) and Leontyev (1974, 1978, 1981), although humans are biological organisms, each human *personality* is the product of social development within the historical mode of life, involving the mastery of tool and sign mediated activity through which they appropriate the social and cultural heritage of their society. Furthermore, both thinkers concluded from their respective studies that the human personality was continually transforming itself through its social activity and does so in its *entirety.* That is to say, thought does not develop without also transforming perception, sensation, memory, and emotion. Indeed, all these things are indivisible elements of human experience that can only be separated from each other analytically, as they often are in psychological studies. This means that feelings and emotions – as we refer to them today in western culture – are every bit as much a cultural and historical product as our forms of cognition and social concepts. They are created in the history of the social relations of a society and are a product of its cultural heritage, forming in symbolically mediated activities and through cultural artefacts, such as literature and music.

The problem in developing an understanding of emotions from the work of these two thinkers is that Vygotsky did not live long enough to fully develop his insights into the role of emotions in psychological functioning, while Leontyev focused primarily on activity and motivation. Although emotions do figure in Leontyev’s writings, the subject appears only briefly and in many ways is lacking. To build a fuller picture of emotion in the cultural-historical framework we will have to turn to other work within this tradition, some of it more recent. I also want to suggest ways forward towards a more fully formed understanding of emotion as a cultural and historical phenomenon, and as part of the socially developed personality considered as a whole.

## Vygotskyan Approaches to the Emotions

Vygotsky is most famous for his work on the development of thinking in children, particularly through their gradual mastery of language at different stages of development, leading to the social and historical formation of the ‘higher mental functions’ that are based in ‘word meaning’ (Vygotsky, 1987a). The assimilation of language through social interactions with others is what transforms the child’s innate mental functions into the higher mental functions, the latter operating according to the laws of cultural and historical development rather than biology. The final stage in the development of the child’s consciousness is concept formation, something which results from its formal education in school, with scientific concepts being the highest intellectual achievement of humankind. However, in the final chapter of *Thinking and Speech* there is the tantalizing comment, frequently quoted, that thought is not born out of other thoughts but ‘has its origins in the motivating sphere of consciousness, a sphere that includes our inclinations and needs, our interests and impulses, and our affect and emotion’ (Vygotsky, 1987a, p. 282). This shows that Vygotsky was interested in more than just the development of different stages in the child’s thinking, being concerned with the development of its consciousness and personality as a whole under the influence of social relationships, language and culture.

Indeed, Bozhovich (1977) has remarked that in the final phase of his thinking in the early 1930s, Vygotsky was troubled by the intellectualism implicit in his theory of consciousness and personality. As a result he devoted the last few years of his life to a study of the emotions and how they are a fundamental aspect of human personality that develop in relationship to the other mental functions and to the child’s social situation, through which it assimilates the social and cultural heritage (Vygotsky, 1994). Despite this, those works of Vygotsky’s that directly address the question of emotion reach no clear conceptualization of it, but instead are largely critiques of existing theories. In a lecture from 1932, Vygotsky (1987b) sees the field of emotion studies at that time as divided by the Cartesian dualism between materialism and idealism, with materialist understandings of the emotions grounded in naturalism – which was then moving from conceptualizing emotions as manifested in the peripheral sense organs towards locating their production in central brain functions – while idealist approaches conceptualized emotions within the subjective or ‘spiritual’ plane. Vygotsky praised psychoanalytic work for its developmental approach, understanding emotion as part of the psycho-dynamics of personality formation through different stages of child development, although for Freud only within specific naturalistic limits. Overall, what Vygotsky seems to be aiming for is a historical materialist approach in which the lower emotions are developed into higher ones through the process of cultural development, and that emotions are never to be conceptualized as separate from or opposed to thought: rather, emotion and thought are found in a different relationship in various types of thinking, such as realistic thinking, ‘autistic’ thinking, or in imagination (Vygotsky, 1987c). In the light of this attempt to overcome the division between materialism and idealism we can see the appeal of Spinoza’s philosophy for Vygotsky, because in opposing Cartesian dualism Spinoza argued for the unity of mind and body in emotional experience, saying that ‘the human mind perceives, not only the affectation of the body, but also the ideas of these affectations’ (Spinoza, 2000, p. 137). For Vygotsky, though, Spinoza’s ahistorical philosophy must have been lacking and perhaps this is why he concludes that although Spinoza attempted to unify causal explanation with the vital meaning of the human passions, this problem awaited its solution (Vygotsky, 1972).^2^

For Bozhovich (1977) and for other scholars writing more recently (Fleer et al., 2017; González Rey, 2012, 2014; Mahn & John-Steiner, 2002), it is Vygotsky’s work on the development of the child in relation to its environment or immediate social situation that is of interest in creating a richer understanding of the role of emotion in the development of personality. Here, Vygotsky wanted to explain how ‘an emotional experience *[perezhivanie]* is always related to something which is found outside the person’ in their social situation, and yet what is represented in this ‘is how I, myself, am experiencing this’ so that ‘all the personal characteristics and all the environmental characteristics are represented in an emotional experience’ (1994, p. 342). The term used here, *perezhivanie,* is a word that is difficult to translate into English and has many possible meanings in Russian but in general is translated as ‘experience’ (Blunden, 2016). However, as Blunden notes, in English the term has passive connotations, as in the sense of living through events that have simply ‘happened’ to us, or as life experience that is suffered or accumulates. In Russian, though, the term has an active meaning as life-changes, episodes, and transformations that we create for ourselves. For Vygotsky the term also indicated how we experience life from the centre of our own personality as a unity or whole, so that our active relationship to others and to the social and cultural world is not primarily cognitive or emotional, based on intellect or perception, or motivated by need or will, but is all these things at once. It is also based in our own personal ‘sense’ – how we understand the meaning inherent in social life on the basis of collective activities and language, but always from our own particular position within it. In terms of emotions, Bozhovich has said that we can draw a general conclusion from this unfinished phase of Vygotsky’s work, that:

…*the development of man’s affective life and needs is basically governed by the same laws as the development of his cognitive functions: they are mediated by socially acquired experience, enter into complex relationships with other functions, and together with the latter form new psychological structures. Furthermore, it may be assumed that affective processes are necessary constituents in the development of cognitive structures as well, without which the study of the human personality would remain one-sided, to say the least (*1977*, p. 17)*.

Emotions, then, are the result of the cultural and historical formation of the human personality and are linked to other indivisible elements of experience, such as need, perception and cognition. Furthermore, in the course of a person’s life, their feelings and emotions will constantly be transformed, taking on new qualities with the changes in their lived experience. As an example, Bozhovich says that love between two human beings ‘is different not only from the sexual drive of animals but also from the love of people living in other historical periods and from the love of people having a very different individual experience’ (1977, p. 20). Even the emotion of being in love, then, which has the most personal sense for each one of us, could not be experienced without its meaning being part of the cultural development of particular societies and embedded at the core of our closest relationships.

From a Vygotskyan perspective, Ratner (2000) has traced a brief but detailed and complex history of the emotion of love between adults in western societies, encapsulated in three periods. First is the period of ‘romantic love’ among the aristocracy during the middle ages; second is the ‘spiritual love’ or rational friendship in the period between the decline of feudalism and the rise of industrial capitalism in the seventeenth and eighteenth centuries; finally there is the period we are living in today, that of ‘modern romantic love’, which is a ‘passionate/sensuous, visceral, spontaneous, irresistible, disorientating feeling that is quickly aroused by personal attributes and physical appearance of another individual’ (Ratner, 2000, p. 12). These feelings are crucial in forming an intimate psychological bond with another person based on shared interests and interpersonal attraction, in which both parties feel they are completed by their union. In each of these periods the shape of people’s interpersonal relations and their feelings for each other are based in the broader economic and power relations of their society. Modern romantic love, for example, reflects the culture of the bourgeoisie and the individualistic nature of their market-based economic activity, which is recapitulated in the individualistic qualities of the modern style of love relations. In turn, the social organisation of activities is represented in concepts that form individual psychological phenomena, intentionality and agency. We can say that the concept of modern romantic love is detailed in cultural works such as art and literary fiction (in novels, plays, songs, and films) and disseminated through the media. Being exposed to these things from a very young age, elements of the concept embeds itself in our psychology as they make sense to us in the active creation of our own lives and relationships.

However, Ratner’s approach has been criticized for being too conceptual as it is centred mainly on cognition, mirroring the intellectualism of the main body of Vygotsky’s work. As such it ignores the bodily component in the experience of emotion, reducing this to a general state of physiological arousal (Holodynski, 2013). To a degree, Ratner is correct to state that any one specific emotion can be associated with various physiological states and, conversely, different emotions can share the same physiological substrate, so that a rise of adrenalin in the nervous system and the corresponding physiological effects like racing heartbeat can be an accompaniment to both fear and excitement. In this sense it is right to say that an emotion cannot be identified by, and therefore be experienced by, the state of physiological arousal alone. But the converse of this is also true, in that many emotions are associated with certain physiological responses and that without these the experience of emotion would lose one of its most vital components and motivating forces. Modern romantic love gains its intensity from its dizzying, disorientating quality and from its components of sexual desire and personal need. But these things do not exist without their symbolic qualities either, so we return to the task of understanding human personality as a unity of dynamic relations between body and mind, cognition and feeling.

In light of this task, Holodynski’s (2013) alternative to Ratner’s theory is no real advance. He integrates Vygotsky’s theories with Leontyev’s ideas, particularly the latter’s view that emotions are inner psychological signals that perform the function of appraising the relationship between the goal of a person’s action (and its outcome) and the motive or need that instigated the action. In the next section of this article I will discuss the implications of this particular reading of Leontyev, stemming from the conflation in translations of his work between the terms ‘object’ and ‘goal’ as the ends of (and motives of) activity. Citing not only Leontyev but also Frijda’s (1986) work in appraisal theory, Holodynski defines emotion as ‘a functional psychological system involving the synchronic interplay of several components and serving to regulate actions within the macrostructure of activity in line with a person’s motives’: within this system the four components that make up an emotion are ‘appraisal, expression, body regulation, and subjective feeling’ (2013, p. 11). However, just as in Frijda’s (1986) flow model, cognitive appraisal is the starting point of the flow of these components, because it is the appraisal of a situation that leads to expressive behaviour and to the internal feelings that create the action readiness for the individual to actively strive toward their goal. But this is a more individualistic cognitive model compared to Ratner’s, even if it allows for bodily feelings as a component in the course of emotion, and it is also narrowly focused on emotion as the motivator and appraiser of goal attainment.

If we wish to consider Ratner’s cognitive model more closely in terms of emotion, it all depends how we define the term ‘concept’ in this regard, something that Ratner does not do himself. It seems to me unlikely that an emotion like modern romantic love is a clearly worked out intellectual concept in the way that a scientific theory is. Indeed, it is part of those concepts that Vygotsky (1987a) called ‘everyday concepts’, such as knowing what it means to be a brother or a sister, a mother or a father. In this light, our own personal sense of an emotion like romantic love is an everyday concept based on the relationships we have been engaged in ourselves or have witnessed, initially within our family and then in wider social circles. As noted above, in modern society ideas about what romantic love is will also be filtered through cultural models provided in the media. Additionally, there is also the question of our personal sense of what and whom we find sexually and romantically attractive. Cultures have in various places and times provided different aesthetics of beauty, yet how this filters into a personal sense of beauty and attractiveness depends upon the interpersonal experiences of the individual.

In his book on romantic love in the modern age, Roland Barthes asks the question of why he finds a particular person attractive: why *this* person and not another? Is it the way ‘a nail is cut, a tooth broken slightly aslant, a lock of hair, a way of spreading the fingers while talking, while smoking?’ (Barthes, 1990, p. 20). Barthes calls these psychic images of the other that so entrance us the ‘image-repertoire’ and claims this is something we find impossible to fully articulate in words. Instead it is articulated by images of the loved one in aspects of their being – a look in the eyes, a way of smiling, youthfulness or maturity, something in the way they move – that answers to our need or desire. If this can be articulated at all, it is, for Barthes, summed up in one word: ‘adorable’. This idea reflects how Vygotsky (1987a) thought that in the internal conversation, which results from the historical development of consciousness through tool and sign mediated activity, we can express to ourselves a thought or a feeling in one single word or image that it would take sentences to express to another, if that would be possible at all. Alternatively, a single word or image can be used to express a whole range of different meanings, internally or externally in dialogue, as in the case Vygotsky cites of the author Dostoevsky who one evening observed a group of drunks expressing a range of thoughts and feelings in the repeated use of a single short noun (Vygotsky, 1987a: 271). This was done not solely through the use of a single word but by the vocal intonation and bodily/facial expressions through which it was uttered, conveying what the drunks were feeling. It is also the case that at times we may struggle, or find it impossible, to articulate in words the thoughts we have as word meanings or image-repertoires, so that ‘words fail us’ and we are left with vague senses or feelings of what we want to express. In these examples we can see two things: that a conceptual thought, or complex ambivalent feelings, can be expressed in a single word or image (or fragmentary images that only attain unity when an object appears that answers to them) as they are rooted in symbolic meaning: and that human consciousness, based as it is culturally and historically in word and (more generally) symbolic meaning, is a unity of both thought and feeling.

The problem in this approach from a Vygotskyan perspective in terms of a theory of emotions, is that if we follow the main body of Vygotsky’s (1987a, 1994) work on child development, we have to assume an early period of ontogeny where the lower mental functions are governed by biological processes that only begin their social development with the gradual mastery of speech, at which point they become subject to cultural laws of development through various different stages. As Arievitch and Stetsenko (2014) say, this leaves Vygotsky’s work partially grounded in some of the naturalistic assumptions about early infancy common in the psychology of his day. But as they also point out, the power of the cultural mediation of activity actually goes back to the ontogenetic roots of early infancy, where there is ‘pre-linguistically mediated meaning making within the initially completely joint and then shared adult–child activities’ (Arievitch & Stetsenko, 2014, p. 218). Central in this early phase is the guiding activity of others, which at first takes the form of embodied joint activities between children and (mainly) adults, where the older participants’ actions intertwine or fuse with the child’s. Later in ontogenesis, ‘the guiding role of others gradually takes on a more distanced and condensed (abbreviated) form represented by activities “crystallized” first in objects (according to their cultural use) and then in symbols and signs’ (Arievitch & Stetsenko, 2014, p. 218).

The child psychologist Daniel Stern has reached similar conclusions in his micro-analytic studies of the interaction between adults and infants, and these are revealing about the early formation of feelings and emotions in joint activities. He illustrates how when an infant begins to cry or shows signs of agitation, its caretakers have no way of understanding why the child is acting in that way. Nevertheless, the caretakers attribute a motive to the child’s actions, in terms of what it may be feeling; they may read into an infant’s cry the emotion of anger or feelings of frustration or discomfort. They then act on this by picking up their baby and taking it in their arms, nursing it, rocking and comforting it, or by feeding it. They also talk to it, even though it cannot understand the words, saying things like ‘it’s okay honey, it’s okay’. As Stern says, at this stage it doesn’t matter what the adult says as they are talking to calm the baby and also themselves, so that what matters ‘is the music and the sound, not the lyrics’ (1990, p. 37). Put in slightly different terms, the meaning is in the music of the utterance not the words. Stern is also convinced that in situations like this very young infants do not know what they feel, what they want, or what is upsetting them. When an infant begins to cry and scream and to wave its arms and legs in agitation, he refers to this as a ‘hunger storm’ that attracts the attention of the caretakers. In the first few months of life, though, the infant is ‘caught in this rich and intense world of the nonverbal, the purely interpersonal, the immediate’ (Stern, 1990, p.52).

The larger point Stern makes in his work is similar to that made by Arievitch and Stetsenko that these early joint activities and interactions are the fabric of meaningful bodily communications between caretakers and infants, which for the child forms the basis upon which it will gradually learn symbolic communication and language. At four and a half months the child also begins to manipulate objects that become a shared ‘topic’ with caretakers. But for Stern (1985) these early joint activities also have another effect, in that they create the ‘affective contours’ between the child and its carers. Being picked up and comforted, having its attention engaged and perhaps distracted from other things, or being neglected, all produce in the child ‘vitality effects’ that become part of its ‘emergent self’. At this stage the vitality effects are not what we would call emotions, but I would venture to suggest a very general line of development that goes something like this; the hunger storm of the infant turns into the temper tantrum of the young child when it cannot get its own way, and this, as the child develops further into symbolic and linguistic communication with its subsequent development of reflexive thought, becomes anger, frustration, dissatisfaction and unhappiness. Similarly, the pleasurable experiences of the infant such as having a hunger storm satisfied by being fed or being positively engaged and loved by a caretaker, turn into the total attachment of the child to its caregivers, which become the capacities for love, care, affection and empathy in the older child and young person. Yet this can also, under various circumstances, lead to the experience of separation, loss, sadness and depression. For me, therefore, it is wrong to suggest that emotions do not correlate to a biological substrate of physiological arousal: instead it is better to say that the vitality effects of the young infant are transformed into various embodied emotions, even though some may share the same or similar vital forms. But the main point I am making here is that what we refer to as feelings and emotions are a product of development and will occur not only as an aspect of the unity of the personality, along with the development of cognition, sensation and need within tool and sign mediated activities; they will also develop as the child and young person’s social relationships change as she passes through the transformations and episodes of her biography.

Before moving on to develop these ideas, it is important that we consider the work of Leontyev, which Roth (2007) has described as the ‘second generation’ of emotion theory in CHAT. This is because Leontyev emphasized how the meaning of objects, which are the focus of thought and emotion, is rooted in social activity.

## Activity Theory: Emotion, Motivation and Need

A. N. Leontyev carried on many of the important elements of Vygotsky’s work, particularly the idea that tools and signs are the objective social medium through which joint human activity is achieved, and that signs are the element of this activity that can be turned towards the self to fashion the inner world of consciousness. However, Leontyev (1974) also emphasised not only how social activity is always objective before it becomes subjective, but also that social activity is oriented to objects that are *produced* in activity, including their symbolic meaning for the social group. For Leontyev, there are two basic characteristics of objective human activity: firstly, activity must be oriented to a real object and, secondly, a mental image of the object is realized through activity as the object takes on a meaning for the individual. This is how consciousness develops as ‘the reflection of reality refracted as it were through the prism of socially developed *linguistic* meanings or concepts’ (Leontyev, 1981, p. 221). As Kaptelinin (2005) has pointed out, there are two words used in the Russian language that are important in understanding Leontyev here: one is *objekt,* which refers to material things existing independently of the mind, and the other is *predmet* meaning the target or content of a thought and action. The latter word, lost in English translation, relates to how Leontyev refers to ‘object’ as its image or meaning in consciousness. It is also in this sense, according to Kaptelinin, that the object of activity becomes its true motive in Leontyev’s theory of personality.

I feel it is important to hang onto this definition of object in Leontyev’s activity theory, as the term is often taken, as it can be in English, as meaning ‘object’ in the narrow sense of a goal or an outcome of activity. Clearly, at times, Leontyev (1974) means to use the term ‘object’ in this way, as when he breaks activity down into three macrostructures; the first being the patterns of social activity we participate in as individuals and which form the basis of our motivations: the second are the specific actions of persons and the goals at which they are aimed: and the third are operations, the means and methods of action, or the way in which actions are performed. In the second macrostructure of activity, actions and goals are clearly linked. However, it is important to note that these macrostructures are not separate aspects of activity but the inner relations within its overall flow. Objects and goals only become motives to actions within a system of social relations and activities, outside of which they would lose their meaning. Activity, then, forms a dialectical relation with the motive, as it is the meaning of activity that creates the motives to act.

It is only in the terms of Marx’s anthropology that we can understand what Leontyev is saying here, because this anthropology reverses the non-Marxist belief (as in appraisal theory in contemporary psychology) that it is individual motives and goals that drive human activity. For Marx, human productive activity has created objects (products) that have changed human needs from biological into historical needs: we can also add to this that natural objects also take on a meaning in activity, as when other human beings have various forms of meaning and importance in our lives. The Marxist Lucien Sève (1978), who Leontyev cites, has shown how this reverses the traditional biological formula in anthropology of human need leading to a product to satisfy it (N/P), into the formula that, historically through production, it is the products of a culture that lead to the creation of need (P/N). In the history of forces and relations of production, new needs are created for the products of human activity. This also reverses the traditional view that need motivates an activity that satisfies that need (N-A-N) and instead it is the activities of socially organised production and their meaning that creates needs or motives that can only be satisfied by activity (A-N-A).^3^ As Leontyev says,

*…human needs are generated by the development of production. After all, production is directly also consumption, which creates need. In other words, consumption is mediated by a need of an object, its perception or its mental presentation. In this, its reflected form, the object appears as the ideal, internally generated motive* (Leontyev, 1978, p. 117).

In the above, we find the broader notion of an object of activity in Leontyev’s work, which I feel allows us to create a more fully formed view of motive, need and, also, of emotion. If we go back to the example of modern romantic love used in the last section of this article and follow Barthes in understanding this emotion as based in the image-repertoire that is created and transformed throughout our lived experience of relationships with others, the object of my desire is another human being, externally objective, who answers to my need in the form of my symbolic perception or mental presentation of them. Something in the body and personality of the other answers to fragments of the image-repertoire, created in my own personal history or biography, which symbolizes my need and desire, motiving me to create an interpersonal and sexual relationship with the other. But this need and desire will also be coloured by emotions, such as longing, yearning, feeling entranced, ‘in love’, and finding the other adorable. If the object of affection proves to be unattainable, then these emotions can turn into anguish, despair, heartbreak, sadness and loneliness. The point here, though, is that an object of a need or motive is not simply a goal, although that is also relevant in terms of the goal of wanting to have a relationship with a loved other: but it is the loved other that is the object, which is also true of many of the other objects of activity, whether they be naturally occurring external objects or those produced by human labour, such as consumer items or works of art. These objects will have their psychic image in human consciousness and be the object of historically developed need, desire, fantasy and emotion. Conceptualizing these aspects of the psyche in this way also provides an opportunity to rethink concepts like ‘desire’ away from their typical Freudian representation – desire or fantasy as internal ideas in consciousness that represent unconscious strivings of the organism operating according to the pleasure principle (Freud, 2005) – and to reconceptualize fantasy and imagination in line with Vygotsky (1987c), as a form of consciousness that develops through the internal use of signs and speech.

Specifically, as to the place of emotions in Leontyev’s work, he says that emotions are internal signals that reflect the ‘relationships between motives (needs) and success, or possibility of success, of realizing the action of the subject that responds to these motives’ (1978, p. 120). Emotions relate to activity more generally because different activities may acquire ‘various and even contradictory emotional colouring’ (Leontyev, 1978, p. 121). This is so because the successful accomplishment of one action does not always lead to positive emotions: in fact, the emotion may be negative if the principle motive of the activity is not realised. This could be the case of a general who wins a battle but remains downcast as he is doubtful about winning the war. Thus, in the complex forms of contemporary society within multiple social activities, human emotions become highly differentiated in terms of objective content and emotional colouring. Sitting in a darkened cinema with a peripheral awareness of my safe surroundings, I may enjoy being scared out of my wits by a horror film about a crazed madman who has broken into a house with an axe, but later on at home in bed with the images from the film still in my mind, it may be less pleasuarble to be disturbed by noises that suggest someone might have broken into the house. In the latter situation, I am terrified for my own safety. Out of this complexity of feeling that occurs only with historical development, Leontyev picks out three classes. First, there are ‘affects’ which are sudden and involuntary, as in the experience of anger ‘overcoming’ us or having a ‘sudden rush’ of excitement. Second, emotions are ‘properly those states – predominantly ideational and situational and the objective feelings connected with them, that is, firm and ‘crystallized’ … in the object of emotional experience’ (Leontyev, 1978, p. 121–122). Third, there are ‘attitudes’ that Leontyev claims are important subjective phenomena in personality formation, although he says nothing more about them.

In addition, another complexifying aspect of emotion in contemporary society is the multimotivational nature of activity and the non-coincidence of motives and goals. In many actions that we undertake, we will not always be aware of having a motive to act, in that we do not account to ourselves as to *why* we are performing that action at that particular moment. Nevertheless, the motive will find a psychic reflection in the emotional colouring of the action, which is to say in its intensity and qualitative character. Sitting in a chair in my study, I may be moved to reach for a book on the shelf that I haven’t read in a long time, or not read at all: why I have reached for the book at this time I’m not quite sure, but my action may be marked by curiosity or interest – a vague desire to read some of it. There are also different levels of motivation to activity. For example, engaging in work activity can be motivated by its social aspect – the fact that we enjoy our relationships with work colleagues and find the collective activity we are engaged in satisfying – or it may have a personal sense for us, in that we enjoy our job and find it rewarding. Thus, for Leontyev (1978), there are ‘social motives’ and ‘sense-forming motives’, the latter being where our activities have a personal sense for us. The meaning of our work may provide a social motive, in that we see being involved in education, health care or business as a social good: yet that also may have a personal sense, for example in the way I enjoy teaching a class, or find reading and writing books and articles intellectually stimulating. The fact that we understand our activities as meaningful shows the connection of our consciousness to culture, whereas personal sense demonstrates that because consciousness ‘belongs to a living individual and is actually part of the system of his activities, it is always passionate’ (Zinchenko, 2009, p. 56). However, not all motives are related to social meaning or personal sense, and Leontyev calls these ‘motives-stimuli’, which are motives such as working for a wage in order to earn money to pay the bills.

In terms of how these ideas of Leontyev’s about emotion might look in practice, Roth (2007) has undertaken a research study on the part that emotion plays in the activities of workers in a salmon hatchery. Here, the actions of workers are coloured by the collective emotion, or mood, in the workplace, with the emotions associated with each person’s actions being dependent on how successfully or unsuccessfully they realize the collective goals of work activity. Positive emotional valence is related to satifaction, accomplishment or expansion of action possiblilties within goal-directed actions, whereas negative emotional valence is liked to failure. An interesting aspect of this study is how, following Vygotsky, Roth links emotion to the workers’ practical reasoning in their jobs and also to their sense of identity. Although this empirical study shows the possibility for further research into emotions using activity theory, Roth’s attempt to create a ‘third generation’ of thinking in regard to emotion on this basis is, for me, less successful. This is because like Holodynski (2013) he attempts to incorporate elements of emotion studies from mainstream psychology into activity theory without a critique of their theoretical basis. Taking Leontyev’s idea that emotions are internal signals that reflect the relationship between motives and goals, Roth understands ‘emotional valence’ as ‘the inner reflection of the current state of organism-environment relation’ (2007, p. 44), so that positive emotional valence results from successful goal attainment and negative valence from its opposite. However, the role of emotional valence is traced to single cell organisms and correlates with their motility and sensibility in the environment. Referencing Damasio (2000), Roth claims that ‘consciousness, motivation and identity are subordinated to orientation and emotional readiness because, from evolutionary and cultural-historical perspectives, the latter predate the former’ (2007, p. 44). However, for the cultural-historical school it is not the case that consciousness, motivation, and identity are subordinated to emotional readiness and orientation because this predates them in evolutionary terms. Rather, the emotions emerge historically just like the other aspects of consciousness and then, within culture, they develop in the ontogeny of each child. When Leontyev (1981) talked about the orientation of higher order animals in the environment according to sensitivity, he was not talking about humans but animals like frogs that have a developed brain and nervous system but lack society and culture. Single cell organisms orient in their environment by irritability, simply responding to elements such as nutrients in their immediate environment. For human beings, however, who develop through activities mediated by cultural-historical instruments like tools and signs, sensitivity breaks down into other components that compose consciousness as a whole, such as perception, feeling, emotion, and intellect, which now direct activity, something that is unknown in the world of other animals.

It is Damasio who believes that single cell organisms have the capacity to have emotions because all they need is ‘a simple perceptual apparatus – a filter to detect the emotionally competent stimulus and the capacity to emote’ (2004, p. 50). This is because, for him, emotions are produced by responses in the organism activated by the emotionally competent stimulus in the environment. In humans and other organisms with a highly developed central nervous system, it is brain mechanisms that produce the emotion, while the stimulus can also come from consciousness in the form of thoughts or images that are emotionally competent. However, for Damasio, consciousness is the result of the brain manipulating images that represent the internal state of the organism, so that the consciousness of an emotion, which he refers to as ‘feeling’, ‘consists of having mental images arising from the neural patterns which represent changes in body and brain that make up an emotion’ (2000, p. 280).^4^ Thus, although Damasio has united body and mind in his version of neuroscience, taking his lead from Spinoza, he is completely oblivious to the fact that the signs and images that create human consciousness are a product of social activity and meaningful communication and do not arise on the basis of brain activity alone. His is a cognitive-behavioural model of the mind, where an emotionally competent stimulus triggers the innate brain mechanisms that produce emotion, which is completely at odds with activity theory. In saying this, I am not suggesting that cultural-historical activity theorists should turn their backs on the discipline of neuroscience, but it is ironic that we have models from within CHAT in the work of Vygotsky, Leontyev, and Luria (1973) that could provide a much more adequate basis for understanding how brain patterns are formed on the basis of social activity, and how the experience of emotions are part of this (Burkitt, 2019).

However, in activity theory a lot of the confusion comes from Leontyev’s own thinking. His notion that emotions are internal signals that reflect the relationship between motives (need) and success of an action is too focused on internal processes. Indeed, I would argue that emotions often take the form of external bodily expressions that are part of social activity and communication. When I see my friend approach me in the street my face may light up with joy and surprise, expressed in culturally embodied signs such as a smile or wave, my internal feelings being totally fused with my external bodily response in the social situation. At other times our bodily expression or vocal intonation may give away a feeling we are trying to conceal. Vygotsky illustrates this by reproducing an excerpt from a play and analyzing it through the ideas of the drama teacher Stanislavskii, who coached actors to find the motive behind their character’s actions within the unfolding drama. The scene opens with Sophia greeting a would-be suitor, Chatskii, with the words, ‘Oh Chatskii, I am glad to see you’: however, something in her intonation or expression tells Chatskii that her emotions about seeing him are ambivalent and so he responds by saying; ‘You’re glad, that’s good. Though, can one who becomes glad in this way be sincere?’ (Vygotsky, 1987a, p. 282). I feel that this example shows how bodily signs, assimilated in interactions with others from our earliest years onward, are read by others as signifying our emotions – such as facial expression or vocal intonation – and, for each of us, are interrelated with the internal conversation in which we realize our emotional response and attempt to regulate it. The point I am making here, however, is that emotions are integral within social interactions and they function in complex ways, not only as internal signals to one’s self but also as signals to others, often ‘spontaneously’ expressed in the moment without full consciousness of our intention. Instead, they give away an intention we would rather have kept hidden.

This leads to a deeper critique of Leontyev’s work on emotions: that he pays too little attention to social relations and *joint* activities, and how the object of an action is not simply a goal, but another person who is its addressee (Sève, in press). Thus, success in a career and attainment of wealth is not just a motive-stimuli for oneself, it can also be an address to another – to the teacher or parent who said you’d never amount to anything; as an opportunity to break into a certain social circle; or as a statement made in the face of the poverty of one’s own upbringing and a way of rewarding the family and friends who supported one’s ambitions. Again, as with the emotion of love, the object of one’s actions are to be seen in the broadest of terms, as an external object in which is crystallized all the many ambivalent emotions one may be feeling in activity, whether the object is a thing which is heavily invested with symbolic meaning or another person for whom one feels deeply. Even if the action has a specific goal in sight, the attempt to achieve it can also have another person or people in view, to whom the action is addressed. This is because activity is not just individual but primarily is collective, an idea which is the basis of Leontyev’s thinking, so that the meaning crystallized in objects is social – something that exists between people – before it takes on personal sense. All the more strange, then, that Leontyev has so little place for others in key elements of his work, not simply as co-participants in collective activity, but as the addressees of all of our activities along with their emotional colourings.

## Social Relations, Personality and Emotions

If we take social relations and joint activities to be primary in society, then emotions are bound up in our social relationships in terms of the everyday concepts we have about them in our culture – what it means to be a mother or father, sister or brother, a lover and/or companion. This is filtered through our own personal sense of these concepts and meanings in our lived experience – how it has felt to be a brother, mother, or lover to the actual people in our lives. These concepts and meanings, however, are fully embodied in the way we feel and think about them, so that emotions always have an evaluative sense. Someone we admire or love is a person whose qualities we hold in high regard, while someone we despise and hate is a person whose characteristics or behaviour has little value for us. As Bakhtin has said, the way we express ourselves in dialogic relations with others is always marked by an emotional-evaluative stance, or what Leontyev might have called an emotional colouring. As Bakhtin says, ‘I experience the object of my fear as fearful, the object of my love as lovable the object of my suffering as oppressive,’ so that ‘lived experience is an axiological position or attitude assumed by the *whole* of myself in relation to some object’ (1990, p. 112–113). When Bakhtin says here that experience is axiological, he is stating that all human experience is meaningful and is about the articulation of values, affecting the way we are oriented to objects in our social world. For example, someone with socialist values, or who highly values other people, may aim for a career in social work, education, or health care, while someone with conservative values may aim to be in business and see making money as the mark of achievement. Thus what Leontyev called social motives, along with the personal sense of our active orientations to others and to objects, will be infused by our values. These also are emotional evaluations of our world and our activities, in that someone who succeeds in business will feel pride and satisfaction in their wealth and status, while the socialist or humanitarian derives satisfaction in giving back to others above financial remuneration.

This also helps to elaborate upon what Leontyev classified as affects, emotions and attitudes. For me, these are not three distinct types of emotion but depend on the way that our emotions are configured in social relations and crystallized in certain objects. For example, a mother may get angry with her misbehaving child in that particular moment without failing for a second to love her. However, it is not that the anger is an affect while the love is an emotion: instead, the love is bound into the long-term relationship between mother and child, thus into their deep reciprocal bond, while the anger at moments of misbehaviour are situational in terms of the here-and-now. That is why the anger only momentarily overrides the love, though not completely. Furthermore, this explains how ambivalent emotions can arise, so that someone we deeply love can also have certain attributes that we dislike or find extremely annoying. In respect of what Leontyev labelled attitudes, I feel this is best addressed in the terms I have elaborated above, in the way that emotions always have an evaluative content linked to the values, beliefs, or ideologies we hold. These things will deeply colour our activities and the way we express ourselves in them in terms of our emotional-evaluative stances. So, we are not dealing with three different classes of emotion here, but the way in which emotion is configured in our social relationships. There are long-term emotions that are bound into our more long-lasting relationships, which are often ambivalent and involve feelings of love and hate, admiration and revulsion, identification and separation towards the object in which they are crystallized: then there are the emotions we feel in particular situations, which are fleeting and may contradict more lasting emotions, although they may not override them except in exceptional circumstances. All emotions, though, will have some evaluative content and so be linked to our values and ideologies, meaning that the different modes of emotional experience will be linked to one another.

Thus, although all emotions will have an evaluative content, this does not mean they are to be understood in an individualistic or cognitive way, as they are in appraisal theory. Instead, if the emotional evaluation is a stance that appears in a particular activity, the evaluation is contained in the emotional act, which is to be considered as a whole – as embodied feeling in thought. As Zinchenko (2009) puts it, emotional evaluation derives from our sense of a situation and involves all the components of consciousness: not just cognition but also the sensory fabric of consciousness, because emotion is present in the perception and expression of meanings in activity and in language, the latter including images, metaphors and symbols. When we sense something within our lived experience it is always a sense of something, such as an action, object, image, or a meaning and, I would add, these things are felt. That is to say, they have the embodied sense of a felt resonance and through metaphorical projection emotional experiences have a location in the body. In English speaking cultures, when we are nervous we feel ‘butterflies in the stomach’ and when we love someone it is felt in the heart. If our love is unrequited, we feel the pain of a ‘broken heart,’ a term that is not just a poetic expression but has real physical consequences such as feelings of being in pain and unable to eat or sleep. It is in this way that social meanings come to have a real, concrete embodied personal sense for us in our experience and how these senses have a meaning that can be shared with others.

The question that remains from Leontyev’s work, however, is to what degree emotions can actually motivate activity? Are emotions purely evaluative or do they have a motivating function in the personality? For Leontyev (1978) one of the ways people deal with the multi-motivational nature of complex social activity, and also with the separation of motives from the goals of activity, is to create a hierarchy of motives, which is understood to be the centre of personality. But how do people do this and what does a hierarchy of motives look like? After all, a motive is not something that exists in itself but is usually considered to be the reason that a person has acted in a particular way, meaning that these reasons can be varied in type. A motive could be an idea, an inclination, a desire, or a need, the latter being most usually associated with motive by Leontyev. But humans have emotional needs, such as the need for an intimate relationship or a satisfying career, and these could be as much of a motive to object oriented activity as any other type of need. Furthermore, we have just illustrated how motives are always linked to values and to social relations, so that the idea of a hierarchy of motives at the heart of the personality seems too self-contained and too static a conceptualization. Perhaps this stems from the fact that, as Stetsenko and Arievitch (2004) point out, Leontyev saw human subjectivity as subordinate to, and originating from, object oriented social activity, and therefore the subject becomes less of a productive, active agent in social life. If we restore this vision and see the expanding cycles of social activity as necessitating a productive self, which also has the capacity to transform itself through its activity – along with its configuration of motives and goals – it means that ‘goals then appear to be dynamic and transformable, potentially feeding not only from but also *into* motives’ (Stetsenko & Arievitch, 2004, p. 492–493, emphasis in original).

In response to Leontyev, Bozhovich (2004) has tried to develop the concept of a relatively stable motivational hierarchy at the heart of the personality, filling it out with the kind of psychological content and developmental process she feels is lacking in Leontyev’s conception. Returning to Vygotsky’s (1994) later works on emotional experience and the child’s relation to the environment, Bozhovich shows how the psychological formation of emotion and need are linked together in the social and historical development of the child’s personality. The emotions that arise in social development are linked to higher needs such as the moral, aesthetic, and intellectual, forming ‘systems structures’ that have new (in the historical sense) psychological content. Thus, complex systemic structures of the personality include moral feelings, consciously set goals and intentions, and personal convictions. The adult personality is then said to be characterized by ‘a set of his own views and attitudes, his own spiritual requirements, and the definite life goals that he strives to attain. All this renders him relatively stable and independent of the environment external to him’ (Bozhovich, 2004, p. 32).^5^

Although Bozhovich’s ideas are an advance on Leontyev’s, showing how the social and psychological development of emotion is linked to that of need, thought, and a moral-evaluative outlook, other aspects of her work remain problematic. First, she takes the same stance as Vygotsky in regarding the early needs and emotions of the child as purely biological until they come to be mediated in tool and sign based activities. However, I have already critiqued this idea here, by showing how preverbal forms of interaction with caretakers actually begin to shape the infant’s affective life from its first days onward. Second, in following Vygotsky’s work on the child’s relation to its environment, Bozhovich’s use of the latter term is too generalized to truly capture the person’s relationship to the social world, which I have indicated here is composed of various different kinds of social relationships in which emotional experience is always located, as well as to various social situations. How a person acts in different situations can be highly variable, showing different selves or sides of the personality in various contexts, so that the personality must be characterized as a dynamic configuration, emphasizing its *relative* stability. For example, my adherence to certain values and my attendant emotional dispositions may lead me to act as a responsible person, but it is unlikely that I will have been able to maintain this, or even seen this as desireable, in every situation. Furthermore, as I said above, people’s more stable emotional orientations are those that are tied into long-term relationships, as are many of their personal characteristics, so that these can change over time as relationships change, but also as one’s emotional needs and motives are transformed. Third, this leads to questions about the notion that motives and other aspects of the personality exist as psychological ‘structures’ or ‘systems’ when, in cultural historical terms, I would suggest it is better to see these as aspects of a dymanic personal configuration, tied into social relationships, social meanings and personal sense, reflected psycholocially as internal dialogues.

In her work on the emotional experience of the child and the development of personality, Bozhovich (2009) has focused on the child’s relationships to others, yet what is missing from this is the dialogic or interactive nature of relationships and how mutual *identification* is at its core. Instead, she has focused on the child’s *position* in social relations and how others will place demands upon it: for example, teachers see the child as a pupil and have educational expectations of performance, whereas the parents may see the child as a helper in the household and educational attainment as secondary. Furthermore, there is the child’s collective, their friends and fellow pupils, who will also have demands, such as to engage in play or other peer group activities. But I would argue that what influences the child most in this array of competing demands is who it *wants to be like,* who it identifies with. If that is with its teachers, then its emotional energies will be directed at education, perhaps wanting to have a career in the future like a teacher or some other professional. If the identification is with one or both parents, then the child may focus on other activities valued by them and want to live up to their expectations and be recognized by them. As Stern (1990) points out, parents have deep wishes, fears, and aspirations for their child, created out of their own past and present experiences, something that can cause problems when the parents’ fantasy does not correspond with who the child is becoming. And then there is the child’s peer group: if the child’s identification is with them, then they may get pulled into activities that dismay their parents and teachers. But the issue here is emotional identification: how the child sees itself in relation to others – who it wants to be like and to please – and thus the place it wants to occupy in school, family, and peer group relationships.

What is of interest here is the reciprocal relation that exists between the person’s identification with others and how emotional experience is at the centre of this. This is of importance not just in terms of the way one feels about others but also in the way one comes to feel about oneself. Again, as Bakhtin shows, the emotions we come to feel about our own self – and through these the whole style of our way of relating to others – is formed in dialogic relations, through which we value each other in various ways. Thus, the way I come to see myself and to feel about myself is initially created…

*…for me by the manifold acts of other people in relation to me, acts performed intermittently throughout my life: acts of concern for me, acts of love, acts that recognise my value. In fact, as soon as a human being begins to experience himself from within, he at once meets with acts of recognition and love that come to him from outside – from his mother, from others who are close to him (Bakhtin, *1990*, p. 49)*.

Unfortunately, the emotional experience we have in life, including the feelings towards our own self, are not always formed in experiences of love and recognition. However, the point I am making here is this: whatever our emotional experience is, it is formed within reciprocal relations and joint activities with others, whether that is the way others author me by their emotional-evaluative acts directed towards me, or by the way I identify with others through my emotional-evaluative stance towards them, wanting to be like them in certain ways. What this demonstrates is that in social relations and activities we are always both the object and the subject of emotional evaluation; it is not just that, as Bakhtin said, we experience emotion subjectively in the way we find the object of our love as loveable, we also find we are the object of emotional-evaluative acts by others, so we feel ourselves to be loveable from the experience of being loved by another or experience the pain of neglect and rejection. In this way, emotions not only colour our activities within our social relations but they also colour the inner conversation we have with ourselves, with our own self-image, and with the image of others. Thus, emotion is like all other psychological phenomena – a product of symbolic and linguistic meaning that, as such, has both a social and historical existence and a private sense for me in terms of the objects that I love or dislike from within my own emotional experience. But this also includes myself, because in dialogical relations I can also take myself as an object of thoughts, images, and feelings so that I come to have emotional-evaluative feelings about my own self within my inner dialogue.

In all these ways emotions are more than just internal signals: they colour all our object oriented social activities and also colour the psychological sense of an object, action, image, or a meaning. This is because emotions are present in all our reflexive thoughts and also in conscious and unconscious experience. One of the great advances of cultural-historical activity theory is that it allows us to think of psycho-dynamics in a radically different way to that found in psychoanalysis. For example, in Leontyev’s (1978) thinking, consciousness and unconsciousness do not oppose one another, as the unconscious also has its roots in objective activity: it is just that for the unconscious the motive for action has a different place in psychic reflection. However, a more extensive and interesting discussion of the unconscious is found in the work of Voloshinov (1976) who argues that the content of the psyche – thoughts, feelings, desires – are presented in the form of symbols, signs, images, and language, all of which have a social meaning stemming from their place in ideology. For him, it is here that ‘motives of behaviour, arguments, goals, evaluations are composed’ and it is here too ‘that arise the conflicts among them’ and repression of certain motives occur (Voloshinov, 1976, p. 83). What this means is that although Freud regarded the censor that stands on the threshold between the unconscious and conscious mind as the agency that stops certain thoughts and desires reaching consciousness, he could never explain where that psychic agency gets the values according to which it performs acts of censorship. How does it know what content to censor and what to permit? As certain behaviours are permitted at some points in history, or in certain cultures, but not in others, and as the psyche has ideological form, then the actions of the censor must be social and ideological. It must be the norms and values of particular societies as refracted in individual consciousness that does the work of censorship and thus creates the conflict in the psyche between conscious and unconscious thoughts, feelings, emotions, and desires. Voloshinov refers to this as official and unofficial consciousness, which mirrors the official or unofficial ideologies in society; the former being those ideologies and forms of activity that are sanctioned in a society, while the latter are those forms of lived ideologies or activities that are disapproved of or banned.

An example of this in terms of emotions can be given through a reconsideration of different forms of modern romantic love. Until recently in many countries of the western world there was in official ideology a normative taboo against homosexual love and against sexual relations between people of the same sex. In England any sexual act between men was made illegal by the state between 1885 and 1967. This meant that romantic and sexual relations between men happened only secretively – in an unofficial underground homosexual culture of meeting places and shared signs – and although relationships between women were not illegal, they were not socially accepted or widely acknowledged. For many individuals this meant that their love was secretive or not acknowledged at all, becoming part of their unofficial consciousness, and, if that love was expressed, it was often accompanied by feelings of guilt and shame. Those who repressed their love found that both the object and the motive of their desire for a person of the same sex was blocked, and instead they remained celibate or took someone of the opposite sex as the object of their love and desire, with varying degrees of success. From a Marxist perspective, Richard Lichtman has reconceptualised the repressed unconscious as populated by certain motives which are ‘constituted as unrealisable’ in society or by ‘aim-inhibited need’, censored by a process in which ‘motive and countermotive have no existence independent of each other. Both are socially constructed out of a process which construes each only in contrast with the other’ (Lichtman, 1982, p. 192). He contrasts this repressed unconscious with a ‘structural unconscious,’ which is where people are aware of a motive but not of its origin, as in the way people in capitalism are aware of self-interest or competitiveness as motives yet see these as ‘natural’ parts of their biological make-up rather than the motives of a social system. An emotion like greed is then to be understood in its proper social and historical context rather than in the sense of biological inheritance and selfish genes.

It is in all these ways, then, that the configuration of the emotions are structured culturally and historically within the lived experience of each person, along with the other aspects of personality – cognition, imagination, fantasy, desire and needs – including those that can be freely expressed and those that are sanctioned.

## Discussion and Conclusions

What I have been arguing and building a case for here, is that emotions are not to be understood as biological aspects of human being but are cultural and historical elements of the human heritage that develop as part of the personality as a whole, along with perception, thought, and need, assimilated in the ontogeny and life experience of each member of society. The emotions we feel are part of the social relations of society, which are manifested in our own personal experience, and these relations are actively made meaningful in the symbols, signs, metaphors and everyday concepts of a culture. The objects of emotional experience are crystallized in relationships and joint activities and they answer to deep seated personal and emotional needs that have developed in our own life history, located in the specific place and time of the culture in which we are raised and create our life trajectory. A final example of the cultural as well of the personal nature of emotion can be seen in the recent work of the historian of emotions, Fay Bound Alberti, who has written a history of loneliness. In this, Bound Alberti (2019) shows how there is little reference to the word ‘loneliness’ in English literature and dictionaries prior to 1800: up to this time there is frequent use of the word ‘lonely’ but this refers only to the state of being singular or separate. It is only after 1800 that the word ‘loneliness’ starts to be used as referring to an emotion – a feeling of being abandoned or bereft of essential social relationships. Interestingly, this is around the same time as Ratner (2000) saw the advent of modern romantic love, and Bound Alberti traces the emergence of loneliness to the same changes in social relations with the increasing dominance of industrial capitalism, the growth of secularism, and the ideology of the individual. Indeed, we could venture to say that loneliness is in some ways a sister to modern romantic love, in the sense that loneliness is often experienced as the absence of significant relationships in one’s life.

However, as I have been arguing here, because emotions can be regarded as everyday concepts in which social relations are recapitulated, this does not mean they are purely cognitive in character: rather, they are better understood as bodily concepts. That is to say, they are felt and sensed within our life experience as much as they are clearly conceptualized and are accompanied at a metaphorical, symbolic level by bodily feelings. Thus, loneliness is often associated with a feeling of emptiness or desolation and, as Bound Alberti says, by physical feelings of coldness, which lead the lonely to pamper themselves by eating comfort food or taking warm baths. This means that although emotions cannot be identified by bodily feelings in themselves, such feelings are a necessary component of emotional experience, signifying that we are feeling something. We *feel* emotion in a bodily sense not because, as some neuroscientists would have it, a brain mechanism has produced an emotion on registering an emotionally competent stimulus that we may, or may not, come to consciously realize as a feeling. In the context of culture and history, consciousness is not a direct representation or idea of what is going on in terms of the homeostatic regulation of the organism. Instead, human consciousness is the creation of symbols, signs and meanings that are integral to culture and object-oriented activity, and it is signs that provide the higher-level organization of brain functions and systems that produce bodily feeling (Luria, 1973; Burkitt, 2019). This begins to happen early in infancy where the vitality effects and natural expressions (such as smiling or crying) of the infant are moulded in the affective contours created by the relationship between the baby and its caregivers. In this way, meaningful social activity restructures the sensory fabric of consciousness right down to the deepest levels of movement, sensation, feeling, intentionality, and need. In later life when we function at these levels we are not always operating with full conscious awareness, but at a level of sign and imagery that only crystallizes in reflexive consciousness when we meet in activity an object that answers to it. This then emerges into consciousness as imagination and fantasy driven by deep-seated emotional needs that are the product of cultural history and personal experience within our own unfolding biography. It is in this sense that we can regard thought as always containing some element of embodied feeling, because consciousness is based in symbolic images and word meanings and is a unity of thought and feeling. Thus affective processes are as necessary a component in the development of cognitive thinking (including fantasy, imagination and, especially in this regard, desire) as are the development of perception, spatio-temporal awareness, and memory.

However, emotions always have an evaluative role to play as part of the dynamic configuration of the personality and are related to moral and ideological values as well as to a person’s needs. All these things play a role in the inner dialogue that we call thinking, a dialogue continually going on between our needs and desires, our moral evaluations and ideological beliefs, as we actively relate to the external social situation in which we are constantly enmeshed, involving object oriented activities with others. These activities are shaped by social relations, yet they also necessarily involve the experience and sense-making activities of each personality. As Vygotsky (1994) said, emotional experience is always located outside the person in their social situation, but it is also about how I experience this, so that all the social and personal elements are contained in it. In any social situation I am not only acting in the present moment: I am doing so with all the weight of my personal experience as I evaluate the situation through my moral and ideological beliefs, through my emotional attachments to the others who are also part of the situation, as we move through the moment into the future-in-the-making. In acting in this way, not all of my intentions might be clear to me: they may come from some repressed or not fully realized source of motivation: they may not as yet be fully crystallized in any object or goal of activity, and, of course, in any type of social activity I am not always fully in charge of the outcome – that may be due as much to others as to my own actions. Yet through this activity my feelings and emotions are always engaged and always in the process of being transformed, along with the changes in my needs and desires. These moments in activity with others form the episodes in which the life-changes and transformations of the personality occur, which are always freighted with emotional meaning and sense. In this productive activity, we transform something of the social world along with some aspect of ourselves, including our own emotional experience, and we set ourselves new goals and find new objects that create for us new needs and desires.

## Footnotes

1 I use the term cultural-historical activity theory (CHAT) here following the work of Stetsenko and Arievitch (2004) who, while recognising the term does not yet have a settled meaning, use it to refer to the project in Soviet psychology developed by Vygotsky, Leontyev, and Luria, and to their co-workers and followers who have sought to advance this project into the present day.

2 For Evald Ilyenkov (1977), a thinker in the tradition of the Vygotsky school, Spinoza was important in conceiving of humans as part of nature, with each individual a ‘thinking body’ that arises on the basis of its activity, but was still a long way from Marx who conceptualized humans as changing nature, along with their own nature, through productive activity.

3 Kaptelinin (2005) loses this historical and dialectical vision of activity, need and motive in Leontyev, claiming that need comes before activity in the latter’s understanding of motivation. The dialectic of activity and motivation (need) is also lost in Kaptelinin’s view that if the object of activity is its true motive, then object and motive of activity mean the same and retaining the two terms is pointless. But the point, for Leontyev, is this: need and motivation is created within historical activity that produces objects for consumption.

4 Damasio is actually much closer to Freud (2005), for whom the ideas that appear in consciousness are representations of the organism’s unconscious strivings operating according to the biological pleasure principle. Indeed, Freud’s topography of the psyche, divided into unconscious, preconscious, and consciousness, also mirrors Damasio’s (2000) idea that emotion emerges as the unconscious production of brain mechanisms, represented as a non-conscious feeling, which is then only fully realized in self-consciousness as ‘feeling the feeling’.

5 This position has been developed more recently by González Rey (2014), who places emphasis on Vygotsky’s notion of subjective sense, claiming that ‘subjective configuration’ is a generative and self-regulating open system of symbolic-emotional units at the heart of the personality that emerges from personal experience.
